# Effects of Eight-Week Supplementation Containing Red Orange and *Polypodium leucotomos* Extracts on UVB-Induced Skin Responses: A Randomized Double-Blind Placebo-Controlled Trial

**DOI:** 10.3390/nu17071240

**Published:** 2025-04-02

**Authors:** Petra Keršmanc, Tina Pogačnik, Janko Žmitek, Hristo Hristov, Olga Točkova, Katja Žmitek

**Affiliations:** 1 VIST—Faculty of Applied Sciences, Institute of Cosmetics, Gerbičeva Ulica 53, 1000 Ljubljana, Slovenia; petra.kersmanc@vist.si (P.K.); tina.pogacnik@vist.si (T.P.); janko.zmitek@vist.si (J.Ž.); 2 Institute of Nutrition, Koprska Ulica 98, 1000 Ljubljana, Slovenia; hristo.hristov@nutris.org; 3 Department of Dermatovenerology, University Medical Centre Ljubljana, Gradiškova Ulica 10, 1000 Ljubljana, Slovenia; olganco@yahoo.com

**Keywords:** *Polypodium leucotomos*, red orange extract, oral photoprotection, minimal erythema dose, erythema reduction, skin pigmentation, UV-induced damage

## Abstract

**Background/Objectives**: Oral photoprotection is gaining attention as a complementary approach to conventional sun protection. This randomized double-blind placebo-controlled study evaluated the effects of an 8-week dietary intervention with a syrup supplement containing *Polypodium leucotomos* extract (PLE), Red Orange Extract (ROE), and vitamins A, C, D, and E on minimal erythema dose (MED), UVB-induced erythema (Δa*), and pigmentation changes (ΔMI). **Methods**: In total, 54 fair-skinned participants (phototypes I–III) were randomized into either the intervention (IP) or placebo group (*n* = 27 per group). MED, Δa*, and ΔMI were assessed at baseline after 2 and 8 weeks of supplementation. **Results**: Throughout the intervention, MED gradually increased, while Δa* decreased in the IP group. While these changes were not statistically significant after 2 weeks, they reached significance after 8 weeks of intervention. By the end of the study, the IP group exhibited a significant 23.8% increase in MED (from 0.447 ± 0.096 to 0.553 ± 0.142 J/cm^2^; *p* < 0.05) and a 46.2% reduction in erythema intensity (from 2.40 ± 0.94 to 1.29 ± 1.04 au; *p* < 0.0001). In contrast, ΔMI in the IP group (from 0.67 ± 0.81 to 0.82 ± 0.96 au) were comparable to those observed in the placebo group, with no significant differences between groups. **Conclusions**: These findings suggest that supplementation with PLE, ROE, and vitamins A, C, D, and E provides systemic photoprotection by enhancing UV tolerance and reducing erythema without affecting tanning response. This study supports oral supplementation as an adjunct to topical photoprotection, with prolonged use potentially yielding cumulative benefits.

## 1. Introduction

The skin serves as a vital interface with the environment, offering essential protection against various external stressors, including ultraviolet radiation (UVR). Ubiquitous in the environment, UV radiation contributes to various skin disorders, including inflammation, degenerative aging, and cancer [1,2]. UVR reaching the Earth’s surface consists of approx. 95% UVA and 5% UVB, each causing distinct skin damage. UVB radiation primarily affects the epidermis, causing direct DNA damage, sunburn, and erythema and is considered the most potent mutagenic component playing a key role in skin cancer development. It triggers inflammation by activating inflammasomes that release cytokines, chemokines, and ROS, leading to sunburn and, if the UV dose exceeds the threshold, triggers apoptosis in keratinocytes, while also causing hyperkeratosis and epidermal thickening to enhance UV protection. It also stimulates melanin production, leading to adaptive melanization (tanning), which protects against further damage [3,4]. UVA radiation, in contrast, penetrates deeper into the skin, affecting both the epidermis and dermis, and contributes to photoaging by damaging major skin components such as the extracellular matrix, connective tissues, and blood vessels, leading to wrinkles and loss of elasticity. Chronic exposure to UVR leads to the accumulation of senescent cells, which release pro-inflammatory cytokines, worsening skin inflammation and contributing to immunosuppression [5].

To counteract damage from sunlight exposure, the skin employs various protective mechanisms including antioxidants, but even moderate UV light quickly exhausts these defenses [6]. While conventional photoprotection strategies—like using sunscreens, protective clothing, and limiting sun exposure—are essential, they have limitations, including inconsistent application, frequent reapplication requirements, and photostability issues, all of which contribute to suboptimal protection in real-world use [7,8,9]. Given these limitations, recent studies highlight the benefits of oral photoprotection, particularly phytochemicals, as a complementary approach to strengthening skin defense against UV-induced damage. While they may not prevent erythema and acute sunburn as effectively as sunscreens, their antioxidant, anti-inflammatory, and immunomodulatory properties help mitigate oxidative stress, DNA damage, and long-term photoaging. Their potential to enhance photoprotection beyond traditional methods underscores a shifting paradigm in the prevention of photoaging and skin cancer [7,8,9,10].

In the case of topical sunscreens, the sun protection factor (SPF) serves as a straightforward and non-invasive way to quantify the photoprotective effects of the product. It represents a way to measure the short-term harm caused by UV rays and is calculated based on a comparison of the minimal erythema dose (MED) required to produce a noticeable erythema on the skin, with treatment to the MED without treatment [9,11]. Similarly, an increase in MED following the use of a nutritional supplement can be used as an indicator of photoprotection in studies evaluating the effectiveness of orally ingested photo protectants [12].

Extracts from fern *Polypodium leucotomos* (PLE) and red-orange *Citrus sinensis* (ROE) represent new promising photoprotective agents. PLE, a source of phenolic compounds such as ferulic, caffeic, chlorogenic, *p*-coumaric, and vanillic acid—which contribute to its biological activity—has been extensively researched for its antioxidant, photoprotective, chemoprotective, anti-inflammatory, and immunomodulatory characteristics [13,14]. Studies on oral PLE intake show its short-term photoprotective effects against UVB and psoralen plus UVA-induced toxicity [15,16], potential to prevent polymorphous light eruption [17,18,19], and ability to suppress UVB-induced erythema [16,20]. ROE, a rich source of anthocyanins, flavanones, hydroxycinnamic acids, and ascorbic acid, has also demonstrated significant photoprotective potential. In vitro studies have shown its antioxidant [21], anti-inflammatory [22], and UVB protective effects [23,24] in human keratinocytes and fibroblasts, with some of these effects—particularly antioxidant and anti-inflammatory activity—also confirmed in vivo. Limited human studies have demonstrated its photoprotective benefits, including prevention of harmful effects of UVR such as UVB-induced erythema [25,26], protection against aging [25], and photoaging [26].

This randomized double-blind placebo-controlled study aimed to assess the effects of an eight-week dietary supplementation with a novel formulation containing *Polypodium leucotomos* extract, red orange extract, and vitamins A, C, D, and E on the minimal erythema dose and UVB-induced erythema in healthy adults. A secondary objective was to evaluate the supplement’s effect on UVB-induced pigmentation (tanning response).

## 2. Materials and Methods

### 2.1. Study Design

The trial utilized a randomized placebo-controlled parallel two-way intervention design to evaluate the efficacy of an 8-week dietary intake of a test product in comparison to a placebo on skin. It was conducted at a single research site.

### 2.2. Study Population

A total of 54 participants were included in the trial. Their eligibility was evaluated based on the following inclusion and exclusion criteria.

Inclusion criteria: Caucasian volunteers aged between 21 and 65 years at the time of the signature of Informed consent form; Signed Informed consent form (ICF); Fitzpatrick skin phototypes I–III; No skin pigmentation disorders; In good health condition; Willingness to avoid a consumption of any food supplements containing red orange extract, polypodium extract, carotenes, or other antioxidants that could interfere with the results during the study; Willingness to avoid the sun, tanning beds, and tanning products on the test area during the study; Willingness to follow all study procedures and keeping a diary during the study (to follow their compliance and palatability); No changes in dietary habits or dietary supplements in last 2 months prior to inclusion; No changes in cosmetic body care routine in last month prior to inclusion on measurement areas; No recent participation in any other similar study; No sun exposure (both natural and artificial) for at least two months before study start on the test area; Absence of sunburn, suntan, scars, or other active dermal lesions on the test area; Color uniformity of the test area (without tattoo, nevi, blemishes, or solar lentigo and without hair).

Exclusion criteria: Pregnancy or breastfeeding or planning pregnancy in the next 6 months (for women); Known or suspected allergy to any ingredient of the tested products or UV radiation; Dermatological problems in the test area or the requirement for annual mole checks by a dermatologist; Pharmacological treatments (both locally or systemically) that could interfere with the results; Use of self-tanning products for at least 2 months before the study start; Medication with photosensitizing potential, drugs, or corticoids in last month prior to study start; Regular consumption of food supplements containing red orange extract, polypodium extract, carotenoids, or other antioxidants or supplements able to induce skin color in last month before inclusion into the study; Any clinically significant history of melanoma or skin cancer (including their immediate family), serious metabolic disease, digestive tract disease, liver disease, kidney disease, hematological disease, or other illness that could interfere with the study; History of severe reactions from exposure to sunlight (i.e., polymorphous light eruption); Any surgery, chemical, or physical treatment that could affect results on the experimental area within the 12 months prior to the study or foreseeing it for the duration of the study; Regular depilation of test area; Planning a hospitalization during the study. Impaired immune system due to autoimmune diseases or use of immunosuppressive medication; Mental incapacity that precludes adequate understanding or cooperation.

All participants gave written informed consent (ICF) before taking part in the trial. Enrolment was conducted consecutively, with participants randomly assigned to either the placebo or test group, consisting of 27 individuals each. The randomization process used a simple method involving computer-generated random numbers created with the ‘RAND’ function in Microsoft Excel for Microsoft 365 MSO (v16.0), ensuring an equal distribution between the two groups.

### 2.3. Study Products and Intervention

The trial was carried out at VIST–Faculty of Applied Sciences (Slovenia) from March to June 2024.

All subjects consumed 25 mL of the syrup once daily for 8 weeks. One study group (IP group) received investigational product IP (daily dose 25 mL: 300 mg Calaguala leaves (*Polypodium leucotomos*) extract; 100 mg red orange (*Citrus sinensis*) fruit extract; 80 mg vitamin C; 6 mg vitamin E; 400 μg vitamin A; 5 μg vitamin D3) and another study group (placebo group) received placebo product without those active ingredients (25 mL: 0 mg Calaguala leaves extract; 0 mg vitamin C; 0 mg red orange fruit extract; 0 mg vitamin E; and 0 μg vitamin A; 0 μg vitamin D3). Other ingredients in both study products were water; sweeteners: xylitol, sucralose; stabilizers: xanthan gum, gellan gum; colors: anthocyanins, caramel color; acid: citric acid; flavor; and preservative: potassium sorbate.

The composition of the investigational product was based on available scientific data supporting the efficacy and safety of each component. The amount of PLE was based on previous human studies demonstrating its antioxidant and photoprotective effects in humans, with consideration of intervention duration [16,20]. The ROE dose reflects that used in trials reporting beneficial effects on UV-induced skin damage and photoaging [25,26]. Vitamin doses align with European Union daily intake recommendations and are commonly included in oral formulations to support skin health and antioxidant defense [27,28]. The formulation was developed to ensure both biological relevance and compliance with safety standards.

The products were packaged in 500 mL white plastic bottles, providing a 20-day supply per bottle. Over the 8-week intervention period, participants received a total of three bottles. They were supplied with a measuring cup that enabled 25 mL dosing. To ensure product stability and safety, participants were instructed to refrigerate opened bottles (2–8 °C).

Both the investigational and placebo products were identical in appearance, taste, and packaging to maintain double-blinding. Neither the participants nor the investigators were aware of group assignments.

Study products in the syrup form were produced by TOSLA d.o.o. (Ajdovščina, Slovenia) under established controlled conditions in line with food regulations.

### 2.4. Assessments

Regular assessments of participants were conducted at three key stages of the study: at baseline (T0), after 2 weeks (T2), and after 8 weeks of supplementation (T8). To determine the minimal erythema dose (MED), the skin was irradiated with 10 gradually increasing ultraviolet B (UVB) doses (10–100%) using an automated erythema tester, Dermalight^®^ 80 MED Tester UVB (310–315 nm; Dr Hoenle Medizintechnik GmbH, Gilching, Germany; UVB 311 nm) at T0, T2, and T8, as described in detail in 2.4.1. MED was evaluated 24 h post-UVB irradiation at each stage, while skin color measurements were taken at each stage before UVB exposure and at 24 h (redness, a*) and 48 h (melanin index, MI) post-irradiation. Participants were instructed to shield the examined area (lower back) from any kind of natural or artificial light and to avoid applying any skincare products to the examined area 12 h before and 48 ± 3 h after UVB application. All measurements were conducted with subjects seated in a controlled environment, maintaining a temperature of 20–25 °C and relative humidity of 40–60%. Assessments began after a 20-min acclimatization period under these conditions.

To ensure protocol compliance, participants kept a diary throughout the 8-week intervention period to log their test product intake, where they also recorded any deviations from the protocol (e.g., missed doses, incorrect dosing…) or adverse events. These diaries were reviewed at T2 and T8. The measuring equipment was regularly calibrated according to the manufacturer’s guidelines to ensure data reliability. The study results were collected between April and July 2024.

#### 2.4.1. Minimal Erythema Dose Determination

Irradiation for the determination of the Minimal Erythema Dose (MED; J/cm^2^) was performed with the automated erythema tester Dermalight^®^ 80 MED Tester UVB (310–315 nm; Dr Hoenle Medizintechnik GmbH, Gilching, Germany; UVB 311 nm) with max. irradiation intensity 8.2 mW/cm^2^ (MED testing). The Dermalight 80 MED tester provides 10 UVB light emission fields with varying dosages ranging from 10% to 100%, which are adjusted by irradiation time. The irradiation time was chosen according to skin phototype (FT) as follows: FT I—74 s (100% dose: 0.607 J/cm^2^); FT II—92 s (100% dose: 0.754 J/cm^2^); and FT III—128 s (100% dose: 1.066 J/cm^2^). FT was determined objectively through colorimetric measurements of the Individual Typology Angle (ITA°) at T0 before irradiation, as a measurement of constitutive pigmentation with FT classification threshold: very light (I) > 55° > light (II) > 41° > intermediate (III) > 28°. We followed the recommended guidelines for constitutive skin color determination [29,30,31,32].

Testing was conducted under standardized conditions. The Dermalight^®^ 80 MED tester was positioned on the lower back (lumbar region) with the emission field directed upward. Participants returned to the study site 24 h post-irradiation for assessment. According to the guidelines [33], the UVB dose received in the first square with perceptible and unambiguous erythema with defined borders filling more than 50% of the exposed area, interpreted 24 h after exposure to UVB, was determined as MED. The entire procedure, including irradiation, color measurements, and MED determination, was repeated 2 and 8 weeks after the initial visit.

#### 2.4.2. Skin Color Measurements: Redness and Melanin Index

The skin color was measured by Colorimeter DSM-4 (Cortex Technology ApS, Aalborg, Denmark), which uses a full visible spectrum color sensor and provides CIE L*a*b* color space measurements with a* values (red-green axis) being an indicator of skin redness according to the Commission Internationale de l’Eclairage [34] as well as measurements of the melanin index (MI), an indicator of skin pigmentation as it corresponds to the skin melanin level [35]. The measurement also gives the ITA index and FT classification.

The skin color measurements were performed on the test area (lumbar region) before irradiation and 24 and 48 h post-irradiation for MED testing at baseline (before intervention), after 2 weeks (14 days), and 8 weeks (56 days) of intervention. Measurements were repeated 4 times on each of the fields in the test area irradiated with 0.8, 0.9 and 1 MED baseline dose and average for each field calculated.

Erythema formation was assessed at all stages (T0, T2, T8) by measuring the change in skin redness (Δa*) induced by UV exposure. This change was quantified as the difference in a* values 24 h after irradiation compared to pre-irradiation levels. Measurements were taken on a skin area exposed to a baseline dose of 1 MED, as higher MED doses used in some previous studies were found to be less sensitive for detecting changes in erythema formation [36].

The Δa* value was calculated using the following equation:

Δa* = a* (24 h post-irradiation) − a* (pre-irradiation).

The UVB-induced skin pigmentation (tanning) was assessed at all stages (T0, T2, T8) by measuring the change in melanin index (ΔMI) induced by UV exposure. This change was quantified as the difference in MI values 48 h after irradiation compared to pre-irradiation levels. Measurements were taken on a skin area exposed to a baseline dose of 0.8 and 0.9 MED and the average was calculated.

The UVB-induced skin pigmentation (tanning response) was assessed at all stages (T0, T2, and T8) as a variation in melanin index (ΔMI, melanin index variation) 48 h post-irradiation on the areas irradiated with 0.8 and 0.9 baseline MED dose and average calculated. Variation in the melanin index was calculated according to the following equation:

ΔMI values: = MI (48 h post irradiation) − MI (pre-irradiation).

The MI is an objective non-invasive measure of skin pigmentation obtained using reflectance spectrophotometry [31,37]. It quantifies the melanin content by analyzing the absorption of specific wavelengths of light by the skin, primarily in the red spectrum centered at 600–700 nm region where melanin is the predominant absorbing chromophore and the interference from hemoglobin is minimal [38]. The ITA° score also correlates with the constitutive skin pigmentation.

### 2.5. Sample Size Calculations

In one of the previous studies with the treatment group receiving red orange extract compared to placebo [25], the percentage of variance in MED explained by the effect of treatment product was 21.7% in the Caucasian subgroup (partial η^2^ = 0.22). Such an amount of explained variance corresponds to the Cohen’s effect size of 0.53. The ability to detect such an effect size with the 80% power at 5% statistical significance level the total sample size of 52 participants is needed (26 per group of equal size). According to the results of previous studies with *Polypodium leucotomos* extract intake, even a larger effect size of at least 25% is anticipated [16,20]. Presupposing a partial η^2^ of 0.25, it was determined that a total sample size of 50 participants was needed to achieve 80% power at a 5% significance level. Accounting for a 10% dropout rate, a total of 54 participants (27 per group) were included in the study.

### 2.6. Data and Statistical Analysis

The measured skin parameters were evaluated by descriptive analysis at T0 (baseline), T2 (after 2 weeks of supplementation), and T8 (after 8 weeks of intervention). To test if the distribution of phototypes between the placebo and IP groups is comparable (i.e., not statistically different), a chi-square test for independence was used, while a comparison of other baseline parameters was performed using a *t*-test. For a comparison of changes within and between groups at different time points, for scale-level dependent variables, a two-way ANOVA with Bonferroni’s multiple comparisons post-hoc test was used. In cases where distributional normality was significantly violated as determined by the D’Agostino-Pearson omnibus normality test, the Mann–Whitney Test for independent samples (intergroup analysis) or Kruskal–Wallis tests (intragroup analysis) was used. The value of MED and other observed parameters at the mid-point/end of the study was treated as dependent variables, treatment vs. placebo group, time point, and their interaction as an independent variable. The significance level was set at 0.05 and analyses were performed using XLSTAT 2021.4.1 (Addinsoft, New York, NY, USA). GraphPad Prism was utilized to generate the graphs (GraphPad Software, Version 9.5.1., Boston, MA, USA). Statistical analysis output was reported as * *p* < 0.05, ** *p* < 0.01, *** *p* < 0.001, and **** *p* < 0.0001.

## 3. Results

A total of 54 healthy male and female subjects, aged between 21 and 65 years, were included in the study and divided into two study groups, 27 in each. Only subjects of Caucasian ethnic origin with skin phototypes I–III were included.

**Figure 1:**
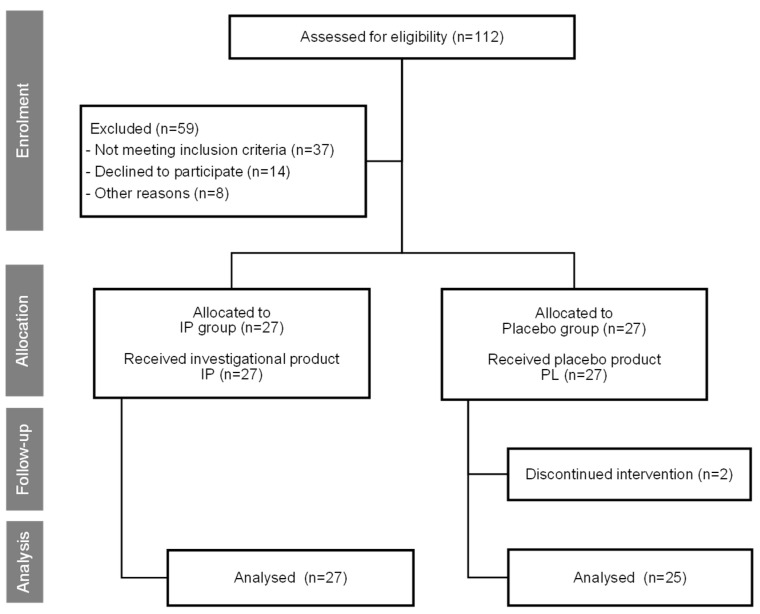
Consolidated Standards of Reporting Trials (CONSORT) flow diagram showing the trial design and subjects’ assignment and progression through the trial.

Out of the 54 subjects enrolled in the study, 52 completed the entire 8-week trial (placebo group: 25 subjects (22 female, 3 male), IP group: 27 subjects (24 female, 3 male). There were 2 drop-outs, both in the placebo group, due to personal reasons. One participant from the IP group was unable to return to the study center for T2 visits due to personal reasons. Both arms demonstrated good compliance with the protocol, as evidenced by the subjects’ diaries of test product intake. There were no adverse effects reported in either of the two research groups, and the supplementation was well tolerated. Trial design and the passage of subjects through the trial are presented in the Consolidated Standards of Reporting Trials (CONSORT) flow diagram in Figure 1.

Participants had a good overall self-reported general health score, with a mean of 4.4 ± 0.6 and no significant difference between the groups (*p* = 0.32). The average age of the participants was 40.6 ± 15.5 years, with no significant age difference between the groups (*p* = 0.75). The mean Body Mass Index (BMI) was 24.7 ± 3.9 kg/m^2^, also showing no significant difference between the groups (*p* = 0.70). Additionally, there were no significant differences in the baseline values of all monitored skin parameters between the groups. Table 1 details the baseline characteristics of participants in each study group.

The distribution of phototypes (FT) was comparable between the groups (*p* = 0.96) as can be observed from Table 2.

Detailed results of the intervention for both groups and results of the statistical analysis are presented in Supplementary Table S1.

### 3.1. Minimal Erythema Dose

**Figure 2:**
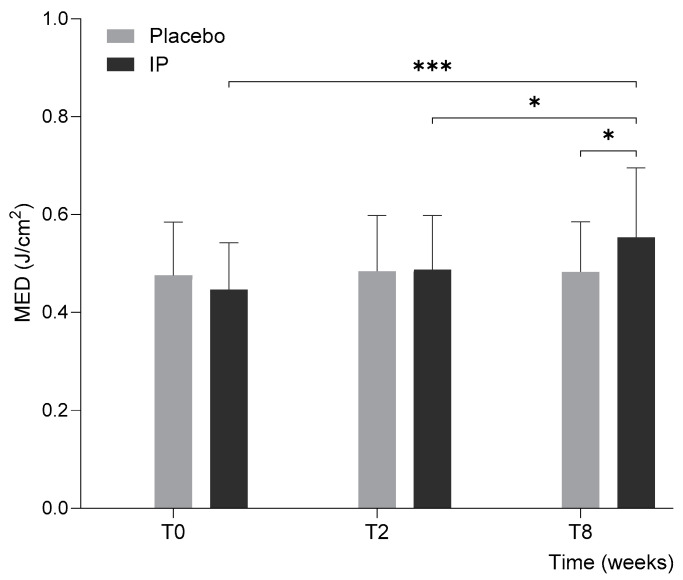
Minimal erythema dose (MED) at baseline (T0) and at follow-ups after 2 (T2) and 8 (T8) weeks of intervention. Error bars represent standard deviation, and the results of intergroup and intragroup statistical analyses are reported above the bars (* *p* < 0.05, *** *p* < 0.001).

The primary outcome, the MED reading, was comparable between the intervention (IP) group (0.447 ± 0.096 J/cm^2^) and the placebo group (0.476 ± 0.119 J/cm^2^; *p* = 0.36) at baseline (Table 1). Figure 2 presents the MED values in J/cm^2^ for both the placebo and the test product (IP) groups at three different time points: baseline (T0), after 2 weeks of intervention (T2), and after 8 weeks of intervention (T8).

In the placebo group, MED values exhibited minimal and non-significant fluctuations over time, reaching 0.484 ± 0.114 J/cm^2^ at T2 and 0.483 ± 0.102 J/cm^2^ at T8. Neither change was statistically significant compared to baseline (*p* = 0.79 and 0.82, respectively).

In contrast, the IP group showed a progressive increase in MED throughout the intervention period, as illustrated in Figure 2, indicating reduced UVB sensitivity. At T2, MED increased by 9.1% (to 0.487 ± 0.111 J/cm^2^) compared to baseline, though this change was not statistically significant (*p* = 0.199 vs. baseline; *p* = 0.92 vs. placebo). However, by T8, MED increased substantially by 23.8% (to 0.553 ± 0.142 J/cm^2^), reaching statistical significance both relative to baseline (*p* < 0.001) and to the placebo group (*p* = 0.028). Notably, the increase from T2 to T8 was also significant (*p* = 0.039), highlighting the importance of prolonged supplementation. These findings demonstrate that supplementation significantly enhanced the skin’s photoprotective function, requiring a higher UVB dose to induce erythema. While short-term use showed initial improvements, only extended supplementation (8 weeks) resulted in a significant reduction in UV sensitivity and increased skin resistance to UV-induced damage.

### 3.2. UVB-Induced Skin Redness

The second outcome aimed to evaluate the impact of the intervention on chromatically determined change in skin redness (Δa*) induced by UV exposure. Δa* was quantified as the difference in a* values 24 h after irradiation compared to pre-irradiation levels at baseline (T0) and after 2 (T2) and 8 weeks (T8) of intervention.

In this instance, a significant difference between the two treatment groups was observed, with erythema formation progressively decreasing in the IP group compared to the placebo group. By the end of the intervention, it was notably reduced in the IP group compared to the placebo group.

**Figure 3:**
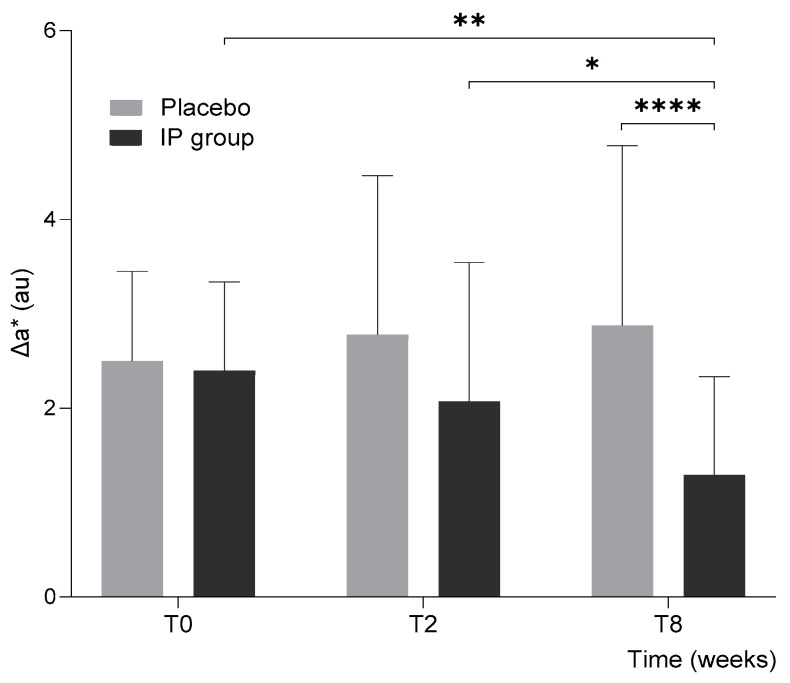
The UV-induced skin redness (Δa*) 24 h after irradiation for the placebo and IP groups at baseline (T0), after 2 (T2), and 8 weeks (T8) of intervention. Error bars represent standard deviation, and the results of intergroup and intragroup statistical analyses are reported above the bars (* *p* < 0.05, ** *p* < 0.01, **** *p* < 0.0001).

As shown in Figure 3, the Δa* values at baseline (T0) were comparable between the two groups (Placebo: 2.50 ± 0.95 au, IP: 2.40 ± 0.94 au, *p* = 0.79). In the placebo group, Δa* values showed minor changes over time, with an 11.2% rise at T2 (2.78 ± 1.69 au) and a 14.8% rise at T8 (2.87 ± 1.90 au), which were not significant when compared to baseline (*p* = 0.47 at T2 and 0.34, respectively).

In contrast, the IP group exhibited a consistent reduction in Δa* values over time throughout the intervention, reflecting a gradual decrease in erythema formation following UVB irradiation. At T2, Δa* decreased by 13.7% (to 2.07 ± 1.47 au), though not yet statistically significant compared to baseline (*p* = 0.39) or to the placebo group (*p* = 0.066). By T8, however, Δa* decreased substantially by 46.2% (to 1.29 ± 1.04 au). This reduction was statistically significant not only compared to baseline (*p* = 0.004) and the placebo group at T8 (*p* < 0.0001) but also to T2 (*p* = 0.040), again highlighting the importance of prolonged supplementation.

These findings demonstrate the effectiveness of the IP product in mitigating UV-induced skin redness and reinforce its role in enhancing skin resilience to UV damage over time.

### 3.3. UVB-Induced Skin Pigmentation

The secondary outcome focused on evaluating the impact of the intervention on a change in cutaneous pigmentation after UVB exposure, e.g., skin tanning response [39]. It was quantified as a change in the melanin index (ΔMI) 48 h after UV irradiation compared to pre-irradiation levels. Measurements were taken at baseline (T0), after 2 weeks (T2), and after 8 weeks (T8) of intervention to assess changes over time, in accordance with the literature each time 48 h after irradiation.

At T0, the ΔMI was comparable in both groups (Table 1, Placebo: 0.54 ± 0.55 au, IP: 0.67 ± 0.81 au; *p* = 0.56) and no important differences in ΔMI between the groups were detected throughout the intervention. In the placebo group, ΔMI increased to 0.69 ± 0.62 au at T2 and to 0.62 ± 0.68 au at T8, representing a 16.2% increase from baseline by T8. Similarly, in the IP group, ΔMI values rose to 0.71 ± 0.96 au at T2 and to 0.82 ± 0.96 au at T8, reflecting a 22.4% increase from baseline by T8. However, these changes were not statistically significant between the groups at any time point (*p* = 0.92 at T2, *p* = 0.38 at T8).

Based on those results, we are unable to confirm that the IP product has a significant impact on the tanning response following exposure to UVB irradiation.

## 4. Discussion

The findings of this randomized double-blind placebo-controlled study demonstrate that a dietary intervention with a novel supplement containing a combination of PLE, ROE, and vitamins A, C, D, and E significantly enhances skin resilience to UVB radiation, particularly after 8 weeks of supplementation. A significant 23.8% increase in MED and a 46.2% reduction in UVB-induced erythema intensity (Δa*) were observed after 8 weeks, indicating a substantial improvement in the skin’s ability to withstand UV-induced damage. Although a shorter 2-week intervention showed a trend toward improvement in MED values (+9.1%) and Δa* (−13.7%); these changes did not reach statistical significance. However, the difference between the 2-week and 8-week results was statistically significant for both MED increase and erythema reduction, underscoring the importance of cumulative effects over time. These results emphasize the necessity of sustained supplementation to achieve the full photoprotective potential of the tested supplement.

Our findings align with previous studies on oral photoprotective agents examining both ROE and PLE. Nobile et al. [25] observed a significant increase in MED in both Asian and Caucasian subjects after 8 weeks of ROE supplementation, whereas no significant change was observed following a shorter intervention. Similarly, Nestor et al. [40] showed a higher incidence of increased MED and reduced erythema after 4 weeks of PLE supplementation compared to placebo, reinforcing the cumulative advantages of sustained use, though the magnitude of these effects was not explicitly quantified. Conversely, Puglia et al. [26] documented a reduction in UV-induced erythema after just 15 days of ROE intake. However, the lack of a placebo group in their study limits direct comparisons with our results. For PLE, additional studies have also demonstrated immediate photoprotective effects. Middelkamp-Hup et al. (2004) found that two oral doses of 180 mg of PLE significantly reduced sunburn cell formation and DNA damage markers, though erythema reduction was only observed up to 2 h post-administration of the second dose [16]. Similarly, Kohli et al. (2017) investigated the acute effects of oral PLE supplementation (480 mg: 240 mg^2^ and 1 h prior to exposure) and found a decrease in UVB-induced erythema intensity and reductions in biomarkers associated with DNA damage, apoptosis (CPDs, sunburn cells), inflammation, and proliferation [20]. The early-phase trends in our study also suggest that short-term intake of both ROE and PLE may provide some photoprotective effects, but long-term intervention is necessary for sustained benefits.

Throughout the intervention, no significant changes in UVB-induced pigmentation were found, as indicated by changes in the melanin index (ΔMI), implying that the intervention did not significantly affect the skin tanning response. These findings are consistent with previous research on ROE supplementation, which reported minimal changes in UVR-induced melanin content measured on dark spots [25,26]. This indicates that the photoprotective benefits of the evaluated supplement may be primarily ascribed to its antioxidant and anti-inflammatory properties rather than increased melanogenesis.

The presented study underscores the photoprotective, anti-inflammatory, and antioxidant benefits of interventions incorporating PLE and ROE, particularly in mitigating UV-induced skin damage. To further enhance these protective effects, vitamins A, C, D, and E were included in the supplement [41], as the synergistic combination of polyphenols and vitamins has been shown to more effectively reduce UV-induced reactive oxygen species (ROS) than individual compounds alone [42].

The mechanisms of action underlying the observed effects are supported by the previous literature, which provides insights into their molecular pathways and physiological impact. Research indicates that PLE, rich in polyphenolic compounds (including caffeic, chlorogenic, ferulic, and *p*-coumaric acids), exhibits strong antioxidant activity, effectively scavenging ROS generated by UV radiation. This helps to prevent oxidative damage to cellular structures, including DNA and lipid membranes [13,43]. It also modulates inflammatory pathways, particularly by inhibiting NF-κB signaling and reducing pro-inflammatory cytokines such as TNF-α and IL-6, which play key roles in UV-induced erythema and skin damage. It has been shown that it also inhibits hyperpigmentation and protects epidermal immune function by preserving Langerhans cells, which are otherwise depleted under UV exposure, stabilizes cellular DNA, and also stabilizes collagen by inhibiting matrix metalloproteinases (MMPs) activated by UV exposure [13,14,44].

Several studies have also examined ROE as a potent systemic photoprotective agent with antioxidant, anti-inflammatory, and anti-photoaging properties. ROE is rich in anthocyanins, flavonoids (e.g., narirutin and hesperidin), hydroxycinnamic acids, and ascorbic acid, all contributing to its antioxidant capacity [45]. In vitro studies indicate that ROE neutralizes ROS and prevents lipid peroxidation following UVR exposure [46] and blocks UVB-induced oxidative stress in keratinocytes, inhibiting NF-κB and AP-1 translocation and preventing cellular apoptosis [23]. Additionally, ROE has been shown to downregulate key inflammatory mediators (IL-6, TNF-α) and modulate MMP activity, preserving extracellular matrix integrity and preventing collagen degradation and photoaging [24,47].

While the results of the presented study are promising, several limitations must be acknowledged. First, the study population was limited to Caucasian individuals, which may limit the generalizability of the findings to other skin types. Additionally, the assessment of photoprotection was based on MED measurements, which primarily reflect tolerance to erythemal doses of UVR, largely influenced by the UVB portion of the solar spectrum. However, this approach does not account for potential effects caused by other wavelengths, such as UVA, visible light, and infrared radiation. Another limitation is the complexity of the tested formulation, which contains multiple active ingredients, making it challenging to determine the individual contribution of each component to the overall effect. Lastly, the long-term safety and efficacy of the formulation require further investigation. Future research should focus on elucidating the molecular pathways involved in the photoprotective effects of the combined formulation and exploring its efficacy in diverse populations and settings. Additionally, studies investigating the optimal dosing and timing of supplementation could further explore its applicability as a photoprotective strategy.

## 5. Conclusions

The findings of this randomized double-blind placebo-controlled study suggest that supplementation with PLE, ROE, and vitamins A, C, D, and E provides systemic photoprotection by enhancing UV tolerance and reducing erythema without affecting the tanning response. These results support the use of oral supplementation as an adjunct to topical photoprotection and as a complementary approach to support the skin’s natural photoprotective mechanisms, with prolonged use potentially yielding cumulative benefits. Beyond their clinical relevance, the findings may also support the development of evidence-based nutraceuticals aimed at improving skin resilience to UV-induced damage. While promising, further studies are needed to confirm these findings across diverse populations and investigate the molecular pathways responsible for the observed effects.

## Figures and Tables

**Table 1:** Demographic and baseline characteristics (mean ± standard deviation (SD)) for study groups at baseline and the interaction between them.

	Group		
	Placebo (*n* = 25)	IP (*n* = 27)	*p*-Value
Age(years)	40.8 ± 15.5	40.3 ± 15.9	0.75
BMI (kg/m^2^)	25.38 ± 4.7	24.4 ± 4.3	0.70
Health score	4.4 ± 0.6	4.6 ± 0.6	0.32
MED (J/cm^2^)	0.476 ± 0.11	0.449 ± 0.097	0.36
Δa* (au)	2.50 ± 0.95	2.40 ± 0.94	0.79
ΔMI (au)	0.54 ± 0.55	0.67 ± 0.81	0.56

**Table 2:** Phototypes distribution by groups.

		Group		*p*-Value
		Placebo (*n* = 25)	IP (*n* = 27)	
		Count (Ratio %)	Count (Ratio %)	
Phototype	I	1 (4.0)	1 (3.7)	0.96
	II	13 (52.0)	13 (48.1)	
	III	11 (44.0)	13 (48.1)	

## Data Availability

The original contributions presented in the study are included in the article/Supplementary Materials; further inquiries can be directed to the corresponding author. Raw data are not publicly available due to institutional policies.

## Supplementary Materials

The following supporting information can be downloaded at https://www.mdpi.com/article/10.3390/nu17071240/s1: Supplementary Table S1: Descriptive statistics (mean ± standard deviation (SD)) for observed parameters in placebo and IP groups at baseline (T0) and at 2- (T2) and 8-weeks (T8) follow-ups, their percentage changes from baseline (Δ%), and results of the statistical analysis.
