# Supplementary material for: Effects of Eight-Week Supplementation Containing Red Orange and Polypodium leucotomos Extracts on UVB-Induced Skin Responses: A Randomized Double-Blind Placebo-Controlled Trial
Source: Nutrients. 2025 Apr 2;17(7):1240. doi: 10.3390/nu17071240 (PMC11990338; doi:10.3390/nu17071240)
Supplement: Supplementary file 1 [file nutrients-17-01240-s001.zip › nutrients-3563937-supplementary.pdf]

Supplementary Table S1: Descriptive statistics (mean ± standard deviation (SD)) for observed parameters in placebo and IP groups at baseline (T0) and at 2- (T2) and 8-weeks (T8) follow-ups, their percent changes from baseline (Δ%) and results of the statistical analysis. The intergroup (IP vs. placebo) statistical analysis is reported near the raw data at each time point, while the intragroup (vs. baseline) statistical analysis is reported near the Δ%. Intergroup and intragroup (vs. baseline) statistical analysis is reported as follows: *p<0.05, ***p<0.001, ****p<0.0001, while intragroup comparison between both follow-ups (T2 vs. T8) is reported as § p<0.05.

Variables	Group	T0	T2	Δ_{T2}%	T8	Δ_{T8}%
MED (J/cm^2)	Placebo	0.476±0.119	0.484±0.114	1.8	0.483±0.102	1.5
	IP	0.447±0.096	0.487±0.111	9.1	0.553±0.142*	23.8***, §
Δa* (au)	Placebo	2.50±0.95	2.78±1.69	11.2	2.87±1.90	14.8**, §
	IP	2.40±0.94	2.07±1.47	-13.7	1.29±1.04****	-46.2
ΔMI (au)	Placebo	0.54±0.55	0.69±0.62	13.8	0.62±0.68	16.2
	IP	0.67±0.81	0.71±0.96	6.0	0.82±0.96	22.4

Legend: MED - minimal erythema dose, Δa* - UVB induced redness formation, ΔMI - UVB induced change of melanin index.
